# 

**Published:** 1911-10-14

**Authors:** 


### BOOKS RECEIVED.

A. C. Fifield.

"Racial Decay." By Commissioner Octavius Charles Beale.

Simpkin, Marshall, Hamilton, Kent and Co., Ltd.

"The Technology of Bread-making." By William Jago and William C. Jago.

### Buildings Contemplated and in Progress.

Arbroath.—The directors of the Infirmary have instructed a special committee to obtain plans and estimates for the proposed reconstruction of the Infirmary buildings. The work is expected to cost between £7,000 and £8,000.

Birmingham.—Two homes for infants are contemplated by the Guardians, and Messrs. Whitwell and Son are architects. Fifteen tenders have been received, the lowest £2,840, the highest £3,284.

Birmingham.—Additions to the Infirmary are contemplated. They include the erection of pathological and bacteriological laboratories, *x*-ray department, and extended accommodation for nurses. Messrs. Ward, of Birmingham, are the architects. The lowest tender is £1,297, the highest £1,729; fourteen were received.

Bradford.—A Local Government Board inquiry has been held concerning an application to borrow £20,500 for the purchase of land and the erection of a Female Tuberculous Sanatorium at Grassington.

Bromley.—The Guardians have had under consideration extensions to the workhouse. It has been decided to proceed with new casual wards at a cost of about £4,000, new receiving wards £2,110, male main building and master's quarters £1,212, clothing and other stores £1,622. A sub-committee was appointed to confer with the architect regarding laundry and boiler house estimated to cost about £7,600.

Carmarthen.—Messrs. George Morgan and Son, architects, Carmarthen, have been appointed architects for the extension of the West Wales Asylum at Carmarthen. Forty acres of ground adjoining the existing buildings have been purchased.

Coventry.—Mr. Frank Cox, of London, has prepared plans and specification for additions to Coventry and Warwickshire Hospital in conjunction with Mr. H. W. Chattaway, of Coventry. Builders desirous of tendering are requested to apply to the secretary. The proposed works include new ward block and operating theatre.

Exeter.—It has been recommended that application be made to the Local Government Board for sanction to borrow £15,500 for a proposed extension of the Council's Sanatorium. Detailed plans and specifications have been prepared by the City Surveyor.

Kilmarnock.—Messrs. John Burnet and Son, of Glasgow, are architects for the additions to the Infirmary to cost about £10,000. The firm is well known in connection with the Glasgow Western Infirmary, and Mr. John Burnet, jun., is the architect for the extension of the British Museum now being completed.

Stockport.—It is proposed to put to architectural competition the design for proposed mental wards at an approximate cost of £30,000.

Watton (Norfolk).—The Wayland Union contemplate the erection of new Infirmary buildings. Mr. H. J. Green, of Norwich, is the architect, and will provide quantities on deposit of £5 5s. Separate tenders are invited for main infirmary buildings and for board-room block and stables.

West Ham.—The Guardians of the Poor have had plans prepared by Mr. F. J. Sturdy, F.R.I.B.A., for additions to the Nurses' Home at the Leytonstone Infirmary. Tenders are now invited, and the necessary particulars may be had on deposit of £5.

### The Week's Novelties.

### THE EUREKA CREPE VELPEAU BANDAGE.

(Vincent Wood, Surgical Appliance Maker, Victoria House, Albion Place, Blackfriars Bridge, S.E.)

We have already reported favourably upon this bandage, which is one of the most economical and durable of the elastic bandages containing no rubber to be found on the market. We have, since testing the samples sent us a year ago, had an opportunity of seeing a number of the "Red Line" quality which have been in use for several months under conditions which are calculated to test very severely the qualities of any bandage. We found the "Eureka" still possessed of its stretching quality, so that the bandage could be used with confidence in rendering a limp hyperæmic (as in Bier's method) without paining the patient, owing to its inelasticity, as so many "elastic bandages" do after they have been used half-a-dozen times. The second quality bandages (blue lines) are slightly cheaper than the Red Line brand, but appear to be little inferior, and are particularly serviceable for out-patient work where a cheap but reliable elastic bandage is desired. Full prices for private and institutional consignments may be obtained on application to the makers at the above address.

### LACTIC CHEESE AND ORGANIC PHOSPHATES.

The progress made by the St. Ivel lactic cheese is at once a success well merited by the manufacturers, and a sign that the general public possesses a fine power of discrimination. From first to last the manufacture of this lactic cheese is conducted on scientific lines. After careful sterilisation of the milk the inoculation with the special culture of the lactic acid bacillus is performed by experienced bacteriologists. The particular point on which stress is laid is that in the St. Ivel lactic cheese all the original *organic* phosphates are retained, as the action of the special culture rapidly converts the curd into a cheese ready for immediate consumption, whereas other cheeses, *e.g.* cheddar, take months or weeks to ripen, and during the process the organic phosphates are converted into inorganic, in which form they are of much less use in building up those phosphorus containing molecules which are vital to the efficient working of the cells of the body. The value, therefore, of St. Ivel lactic cheese from the dietetic standpoint is both relatively and absolutely increased, and the public may have every confidence in the products of the most hygienic and carefully managed factory at Yeovil.

### A TEA FOR INVALIDS.

China tea is known to be the tea of connoisseurs. It was the tea drunk by Dr. Johnson at the rate, if we remember, of about twenty cups a night. It is subtler than even the best Ceylon, and we believe this subtlety to represent in practice a cup "less strong"—that is, less impregnated with tannin. These considerations have led Davison, Newman and Co., of 14 Creechurch Buildings, Leadenhall Street, E.C., to claim their "old-fashioned China tea" as suitable for invalids. It is very nice, even to so exquisite a discriminator as the man who has trained his palate on Lapsang Souchong—with a graduated mixture of Oolong flavouring tea in it. The average China tea drinker, indeed, will be glad that the "old-fashioned" China tea has not the other's smoky flavour. It is the sort of China tea, in fact, which converts the Ceylon tea-drinker.

The Brewers' Exhibition.—The thirty-third annual trade gathering in connection with Brewers, Maltsters, Distillers, Mineral Water Manufacturers, etc., will open at the Royal Agricultural Hall, Islington, N., on Saturday, October 14, and remain open till October 20. No efforts have been spared to make the show an attractive one, and the display of machinery and appliances will be found in every way up-to-date, and of equal interest to a large circle that lies outside the actual trade itself. The Malting Barley Competition, held in connection with the exhibition and market, has a value all its own, if only from the fact that farmers have been spurred on to obtaining better seed and devoting more care to barley cultivation. The competitions for hops, beers, cider and perry, Colonial wines and temperance beverages continue to be popular, the number of entries in each section being reported as highly satisfactory. The important function of judging in these various contests has necessitated an accession of strength, the gentlemen selected to adjudicate upon the samples submitted being well qualified for the task. The railway companies are affording the usual facilities in the way of excursion trains to London from all parts of the country.

